# Supplementary material for: A unified hypothesis-free feature extraction framework for diverse epigenomic data
Source: Bioinform Adv. 2025 Mar 8;5(1):vbaf013. doi: 10.1093/bioadv/vbaf013 (PMC11897706; doi:10.1093/bioadv/vbaf013)
Supplement: vbaf013_Supplementary_Data [file vbaf013_supplementary_data.pdf]

Supplementary for “A unified hypothesis-free feature extraction framework for diverse epigenomic data”

Ali Tuğrul Balcı, Maria Chikina

1 Methods

The L0-segmentation problem for noisy observations y_1, y_2, ..., y_N defines the following maximization problem:

$$\max_{\beta_i} \sum_{i=1}^{N} e\left(y_i, \beta_i\right) - \lambda \sum_{i=2}^{N} \|\beta_i - \beta_{i-1}\|_0 \tag{1}$$

e is a concave, differentiable function derived from a parametric distribution family such as Gaussian, Poisson, Binomial. If L_0 norm is swapped with L_1 norm in the problem (1), which converts the problem into fused lasso signal approximation (FLSA), the expression delineates the log-likelihood of an Hidden Markov Model (HMM). From this perspective, β_is are the latent variables that define the different emission distributions, $P\left(y_i|\beta_i\right)$. The transition distribution for a state, $P\left(\beta_{i+1}|\beta_i\right)$, is Laplace distribution with the location parameter, β_i, and the scale parameter, λ. This problem can be solved with Viterbi algorithm in $\mathcal{O}\left(n^2\right)$ time and with Johnson's algorithm in $\mathcal{O}\left(n\right)$ time. However, having L_0 norm instead of L_1 norm renders the objective (1) a difficult case in terms of representing it as likelihood of an HMM. The problem with L_0 norm is the regularization part of the expression (1) no longer defines an exponential probability distribution for the state transitions. Yet, Johnson was successful in extending the algorithm that solves FLSA to L_0-segmentation problem. The dynamic algorithm solves the objective (1) in $\mathcal{O}\left(n^2\right)$ complexity. Fortunately, the algorithm exhibits $\mathcal{O}\left(n\right)$ complexity in applications to various types of biological data.

We will adopt Johnson's approach to solve the maximization problem (1). The first step is to

divide the objective into smaller objectives as follows:

$$\begin{aligned}
&\max_{\beta_N}\left(e\left(y_N,\beta_N\right)+\left(\max_{\substack{\beta_i\\ i=1,\dots,N-1}}\sum_{i=1}^{N-1}e\left(y_i,\beta_i\right)\right.\right.\\
&\qquad\left.\left.-\lambda\sum_{i=2}^{N}\|\beta_i-\beta_{i-1}\|_0\right)\right)\\
f_N(\beta_N) &= \max_{\substack{\beta_i\\ i=1,\dots,N-1}}\sum_{i=1}^{N-1}e\left(y_i,\beta_i\right)\\
&-\lambda\sum_{i=2}^{N-1}\|\beta_i-\beta_{i-1}\|_0\\
&-\lambda\|\beta_N-\beta_{N-1}\|_0\\
f_{N-1}(\beta_{N-1}) &= \max_{\substack{\beta_i\\ i=1,\dots,N-2}}\sum_{i=1}^{N-2}e\left(y_i,\beta_i\right)\\
&-\lambda\sum_{i=2}^{N-2}\|\beta_i-\beta_{i-1}\|_0\\
&-\lambda\|\beta_{N-1}-\beta_{N-2}\|_0\\
&\vdots\\
f_2(\beta_2) &= \max_{\beta_1} e\left(y_1,\beta_1\right)-\lambda\|\beta_2-\beta_1\|_0
\end{aligned}$$

To clarify, we can simplify the above functions f_N, f_{N-1}, ..., f_2 as:

$$\begin{aligned}
&\max_{\beta_N}\left(e\left(y_N,\beta_N\right)+f_N(\beta_N)\right)\\
f_N(\beta_N) &= \max_{\beta_{N-1}} f_{N-1}(\beta_{N-1})\\
&+e\left(y_{N-1},\beta_{N-1}\right)-\lambda\|\beta_N-\beta_{N-1}\|_0\\
f_{N-1}(\beta_{N-1}) &= \max_{\beta_{N-2}} f_{N-2}(\beta_{N-2})\\
&+e\left(y_{N-2},\beta_{N-2}\right)-\lambda\|\beta_{N-1}-\beta_{N-2}\|_0\\
&\vdots\\
f_2(\beta_2) &= \max_{\beta_1} e\left(y_1,\beta_1\right)-\lambda\|\beta_2-\beta_1\|_0
\end{aligned}$$

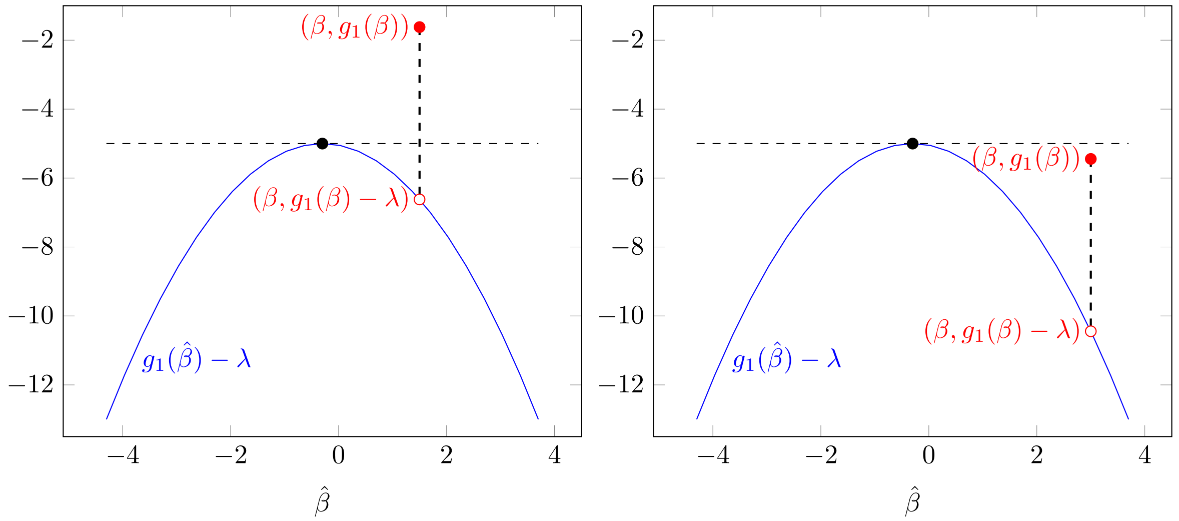

Figure S1: The geometry of $g_1(\hat{\beta}) - \lambda\|\beta - \hat{\beta}\|_0$. Left, $g_1(\gamma) - \lambda < g_1(\beta)$ and thus $\beta_i^*(\beta) = \beta$. Right, $g_1(\gamma) - \lambda > g_1(\beta)$ and thus $\beta_i^*(\beta) = \gamma$.

This can be even further simplified with the help of additional functions:

$$\begin{aligned} g_1(\beta) &= e(y_1, \beta) \\ \beta_i^*(\beta) &= \text{argmax}_{\hat{\beta}}\, g_{i-1}(\hat{\beta}) - \lambda \left\|\beta - \hat{\beta}\right\|_0 \\ f_i(\beta) &= \max_{\hat{\beta}} g_{i-1}(\hat{\beta}) - \lambda \left\|\beta - \hat{\beta}\right\|_0 \\ &= g_{i-1}(\beta_i^*(\beta)) - \lambda \left\|\beta - \beta_i^*(\beta)\right\|_0 \\ g_i(\beta) &= e(y_i, \beta) + f_i(\beta) \end{aligned}$$

Notice that once the forms of β_i^* are known, we can easily solve the optimization problem by going "backward" following β_i^*s. Simply, if we know β_N, the solution of $\text{argmax}_\beta\, g_N(\beta)$, the solution is,

$$\beta_i = \beta_i^*(\beta_{i+1}) \text{ for } i = N-1, N-2, ..., 1.$$

This algorithm is called backtracking.

The key to finding the form of $\beta_i^*(\beta)$ is to realize that $g_{i-1}(\hat{\beta}) - \lambda \left\|\beta - \hat{\beta}\right\|_0$ is λ less than $g_{i-1}(\hat{\beta})$ everywhere except for when $\hat{\beta} = \beta$ as illustrated in Figure S2. Assuming $\gamma = \text{argmax}_{\hat{\beta}}\, g_{i-1}(\hat{\beta})$ then $\beta_i^*(\beta)$ is defined as

$$\beta_i^*(\beta) = \begin{cases} \beta & g_i(\gamma) - \lambda < g_i(\beta) \\ \gamma & \text{otherwise} \end{cases}$$

For the first step, because e_1 is concave, g_1 is also concave. This implies that the maximum value and where it is attained can be calculated easily. Once the point where the maximum is attained is known, let it be defined as γ, we can find the point/points where g_1 is equal to $g_1(\gamma) - \lambda$. For a one

dimensional concave function, we may have one point or two points that satisfy $g_1(\beta_\pm) = g_1(\gamma) - \lambda$. If they exist, β_- satisfies $\beta_- < \gamma$ and β_+ satisfies $\gamma < \beta_+$. With the assumption that the domain of e_1 is (a, b), the region where $g_1(\gamma) - \lambda < g_1(\beta)$ is either (a, β_+), (β_-, b) or (β_-, β_+). If we define this region as E_1 and define its complimentary as F_1, then the definition of β_1^* is

$$\beta_1^*(\beta) = \begin{cases} \beta & \beta \in E_1 \\ \gamma & \beta \in F_1 \end{cases}$$

Now we will first take a look at the forms of f_i and g_{i+1}.

Based on the definition of $\beta_i^*(\beta)$, the definition of $f_i(\beta)$ becomes

$$f_i(\beta) = \begin{cases} g_i(\beta) & g_i(\gamma) - \lambda < g_i(\beta) \\ g_i(\gamma) - \lambda & \text{otherwise} \end{cases}$$

$f_i(\beta)$ is a "flooded" version of $g_i(\beta)$. The value of the function, $g_i(\beta)$ within the regions where $g_i(\gamma) - \lambda < g_i(\beta)$ is mapped to the constant, $g_{i-1}(\gamma) - \lambda$. In other words, those regions are flooded. Thus, $g_{i+1}(\beta)$, for the next step, is defined as

$$g_{i+1}(\beta) = \begin{cases} e_{i+1}(\beta) + g_i(\beta) & g_i(\gamma) - \lambda < g_i(\beta) \\ e_{i+1}(\beta) + g_i(\gamma) - \lambda & \text{otherwise} \end{cases}$$

Even though f_i and g_{i+1} are not concave, they consist of concave pieces. For $i = 2$, $f_2(\beta)$ is

$$f_2(\beta) = \begin{cases} e_1(\beta) & \beta \in E_1 \\ e_1(\gamma) - \lambda & \beta \in F_1 \end{cases}$$

and thus g_2 is

$$g_2(\beta) = \begin{cases} e_2(\beta) + e_1(\beta) & \beta \in E_1 \\ e_2(\beta) + e_1(\gamma) - \lambda & \beta \in F_1 \end{cases}$$

, both f_2 and g_2 are illustrated in Figure S2.

Assuming both β_- and β_+ exist, we see that g_2 is concave over partitions $(a, \beta_-]$, $E_1 = (\beta_-, \beta_+)$, and $[\beta_+, b)$. For $i > 1$, the β_i^*, f_i, and g_{i+1} can be calculated the same way the functions in the first step are calculated except for the later steps, every partition must be iterated over to find global maximum, γ, and to partition the domain by finding where the function value is less than $g_i(\gamma) - \lambda$, in other words flooded regions. This is why the algorithm is $\mathcal{O}(n^2)$ complexity in theory. The partitions can increase linearly at each step. However, this doesn't happen in practice. Whole partitions often get flooded, leaving fewer partitions for the next step.

This analysis yields a blueprint for an algorithm to solve the objective (1). In the forward pass, the algorithm calculates the global maximum and the partitions at each step. Once it reaches g_{N+1}, it calculates $\beta_N = \text{argmax}_\beta\, g_N(\beta)$. Then in the backward pass, it computes $\beta_i^*(\beta_{i+1})$ where β_{i+1} is the solution at step $i + 1$. In other words, the algorithm checks if β_{i+1} is in a flooded partition, if it is then assigns γ_i to β_i where $\gamma_i = \text{argmax}_\beta\, g_i(\beta)$, otherwise it assigns β_{i+1} to β_i.

In the next two section, the solution of (1) for when $e_i(\beta)$ is derived from Poisson and binomial distributions is given. Particularly, we will show the forms of e_i derived from the two distribution and how to calculate the maximum value and partitions from these forms of e_i.

1.1 Poisson Case

Poisson probability mass function is given as

$$f(k;\lambda) = \frac{\lambda^k e^{-\lambda}}{k!} \tag{2}$$

Log transforming (2) results in

$$\log f(k;\lambda) = k\log\lambda - \lambda - \log k!$$

By fitting count data for λ with L_0 regularization, we can cluster count data into contiguous segments, each corresponding to the same λ (mean / variance). One possible definition for the error term is

$$y_i \log\beta - \beta$$

By omitting the constant $\log k!$. The domain of this function is from 0 to infinity. It is much easier to work with a function with a domain from $-\infty$ to ∞, because otherwise 0 is an edge case where special care should be taken. Therefore, we will apply $\beta = e^x$ transformation and use the alternative form for $e_i(\beta)$

$$e_i(x) = y_i x - e^x$$

The maximum point of $e_i(\beta)$ can be found as follows

$$\begin{aligned}\frac{de_i(x)}{dx} &= y_i - e^x\\ 0 &= y_i - e^\gamma\\ \gamma &= \log y_i\end{aligned}$$

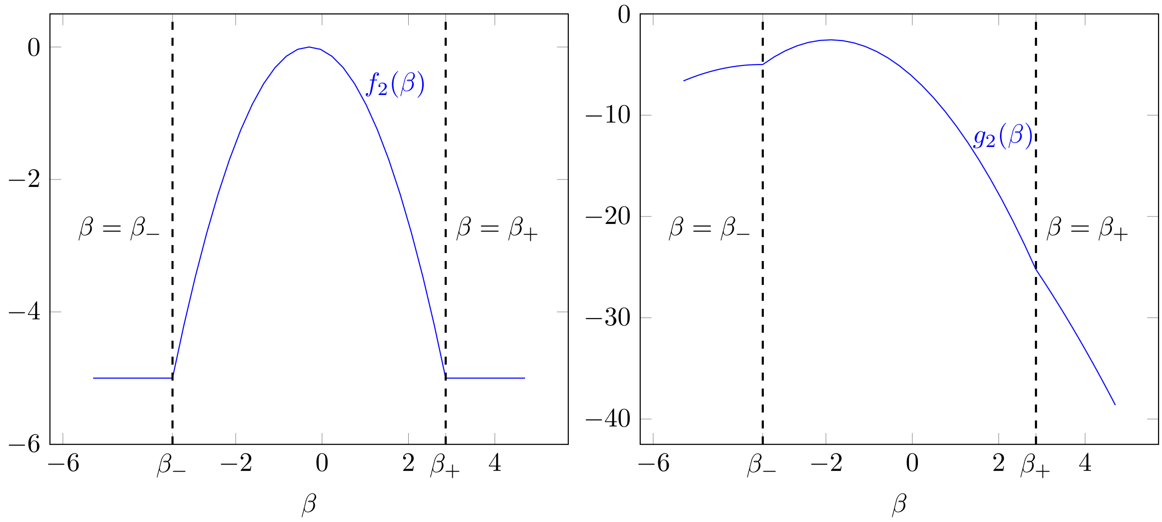

Figure S2: Left, f_2 is shown. When $g_1(\gamma) - \lambda > g_1(\beta)$, f_2 is constant at the value, $g_1(\gamma) - \lambda$. When $g_1(\gamma) - \lambda < g_1(\beta)$, $f_2(\beta) = g_1(\beta)$. Right, $g_2(\beta) = f_2(\beta) + e(y_2, \beta)$ where $y_2 = 3.5$ is shown. g_2 is no longer a concave function but the segments are separately concave.

After the maximum point is found, the partition ends can be calculated as

$$
\begin{aligned}
c &= ax + be^x \\
-ax + c &= be^x \\
-a\left(x - \frac{c}{a}\right) &= be^x \\
-\left(x - \frac{c}{a}\right)e^{-x} &= \frac{b}{a} \\
-\left(x - \frac{c}{a}\right)e^{-\left(x - \frac{c}{a}\right)} &= \frac{b}{a}e^{\frac{c}{a}} \\
-x + \frac{c}{a} &= \mathcal{W}\left(\frac{b}{a}e^{\frac{c}{a}}\right) \\
x &= \frac{c - a\mathcal{W}\left(\frac{b}{a}e^{\frac{c}{a}}\right)}{a}
\end{aligned}
$$

Two real solutions of this equation are

$$
\left(\frac{c - a\mathcal{W}_{-1}\left(\frac{b}{a}e^{\frac{c}{a}}\right)}{a}, \frac{c - a\mathcal{W}_{0}\left(\frac{b}{a}e^{\frac{c}{a}}\right)}{a}\right)
$$

If $y_i = 0$, then we have one solution which is $\log\left(\frac{c}{b}\right)$.

1.2 Binomial Case

Binomial probability mass function is

$$
f(k, n; p) = \left(\begin{array}{c} n \\ k \end{array}\right) p^k (1-p)^{n-k}
$$

Log transforming it results in

$$
\log f(k, n; p) = k \log p + (n-k)\log(1-p) + \log\binom{n}{k}
$$

We will fit parameter p for given n and k vectors. For our specific aim, ns are the list of coverage and ks are the number of methylated reads. Thus the error term for this problem turns out to be

$$
y_i^1 \log\beta + \left(y_i^2 - y_i^1\right)\log(1-\beta)
$$

As we did in the Poisson case, we apply a transformation to convert the domain into $(-\infty, \infty)$. The particular transformation is $\beta = \frac{1}{1+e^{-x}}$. Finally, the form e_i takes for this problem is

$$
e_i(x) = y_i^1 x - y_i^2 \log(1 + e^x)
$$

The maximum point of e_i, γ, can be found as follows

$$
\begin{aligned}
\frac{de_i(x)}{dx} &= y_i^1 - y_i^2 \frac{e^x}{(1+e^x)} \\
0 &= y_i^1 - y_i^2 \frac{e^x}{1+e^x} \\
y_i^2 e^x &= y_i^1 \left(1+e^x\right) \\
e^x &= \frac{y_i^1}{y_i^2 - y_i^1} \\
x &= \log\left(\frac{y_i^1}{y_i^2 - y_i^1}\right)
\end{aligned}
$$

Solving $0 = ax - (a+b)\log\left(1+e^x\right) + c$ turns out to be a difficult problem. Therefore, we will use an approximation instead of the exact solution. First we will get an approximation for each of the possible two roots and then we will refine these approximations via Newton's method. To find approximations for the roots, the original problem $a\log\beta + b\log\left(1-\beta\right) + c = 0$ is more useful because the domain of the function $a\log\beta + b\log\left(1-\beta\right)$ does not span the whole $\mathbb{R}$. In other words, we know that one solution is closer to 0 and the other one is closer to 1.

First consider the larger root, nearby 1. We can apply a Taylor expansion to $f\left(\beta\right) = a\log\beta$,

$$
\begin{aligned}
f\left(\beta\right) &\approx a\left(\beta - 1\right) \;\; (\text{near } \beta = 1) \\
a\log\beta + b\log\left(1-\beta\right) + c &\approx b\log\left(1-\beta\right) - a\left(1-\beta\right) + c
\end{aligned}
$$

Now, this approximation can be solved as follows

$$
\begin{aligned}
0 &= b\log\left(x\right) - a\left(x\right) + c \\
\frac{a}{b}x - \frac{c}{b} &= \log\left(x\right) \\
e^{\frac{a}{b}x} e^{-\frac{c}{b}} &= x \\
e^{-\frac{c}{b}} &= x e^{-\frac{a}{b}x} \\
-\frac{a}{b} e^{-\frac{c}{b}} &= -\frac{a}{b} x e^{-\frac{a}{b}x} \\
\mathcal{W}\left(-\frac{a}{b} e^{-\frac{c}{b}}\right) &= -\frac{a}{b} x \\
-\frac{b}{a}\mathcal{W}\left(-\frac{a}{b} e^{-\frac{c}{b}}\right) &= x \\
-\frac{b}{a}\mathcal{W}\left(-\frac{a}{b} e^{-\frac{c}{b}}\right) &= 1 - \beta_1 \\
\beta_1 &= 1 + \frac{b}{a}\mathcal{W}\left(-\frac{a}{b} e^{-\frac{c}{b}}\right)
\end{aligned}
$$

We can transform this as $x_1 = \log\left(\beta_1\right) - \log\left(1-\beta_1\right)$ and use Newton's method to obtain a tight approximation for the root near 1. The same procedure can be applied to obtain an approximation

for the root near 0:

$$\begin{aligned}
f(\beta) &= b\log(1-\beta) \\
f(\beta) &\approx -b\beta \text{ (near } \beta = 0) \\
a\log\beta + b\log(1-\beta) + c &\approx a\log\beta - b\beta + c \\
0 &= a\log\beta_0 - b\beta_0 + c \\
\beta_0 &= -\frac{a}{b}\mathcal{W}\left(-\frac{b}{a}e^{-\frac{c}{a}}\right)
\end{aligned}$$

We can transform this approximation and use Newton's method as we did for the root near 1.

2 Supplementary Figures

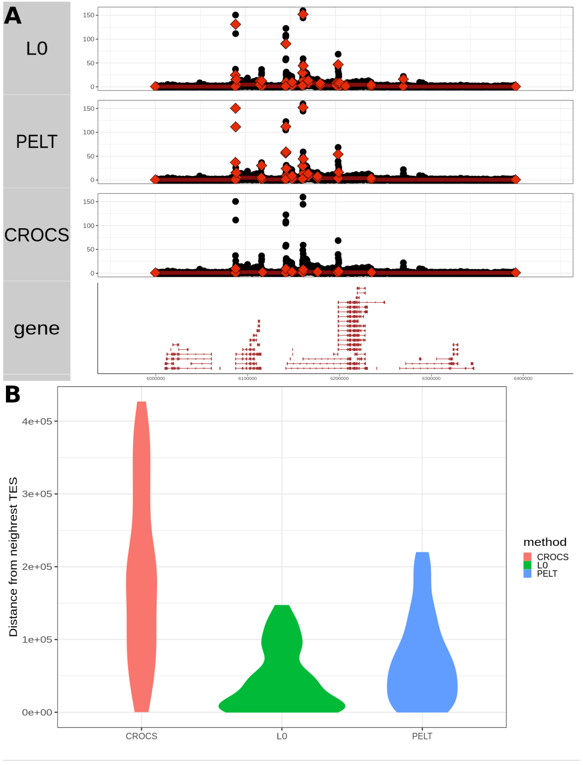

Figure S3: **A**: Illustration of segmentations of RNA Pol II using three different methods. CROCS, PELT, L_0 have identified 13, 24 and 24 segments respectively. **B**: Distribution of distances from 148 transcription end sites identified within the 3Mb long genomic region starting from eight millionth nucleotide in chromosome 12 of Hg38 to the nearest breakpoints identified by one of the methods from a RNA Pol II assay. The hyper parameters for PELT and L_0 are adjusted so that both algorithms produce the same number of break points; for the figure both algorithm produced 122 breakpoints. CROCS adjusts hyper parameters itself, and outputted three different segmentations for this segment. We evaluated the finest segmentation which has 9 breakpoints.

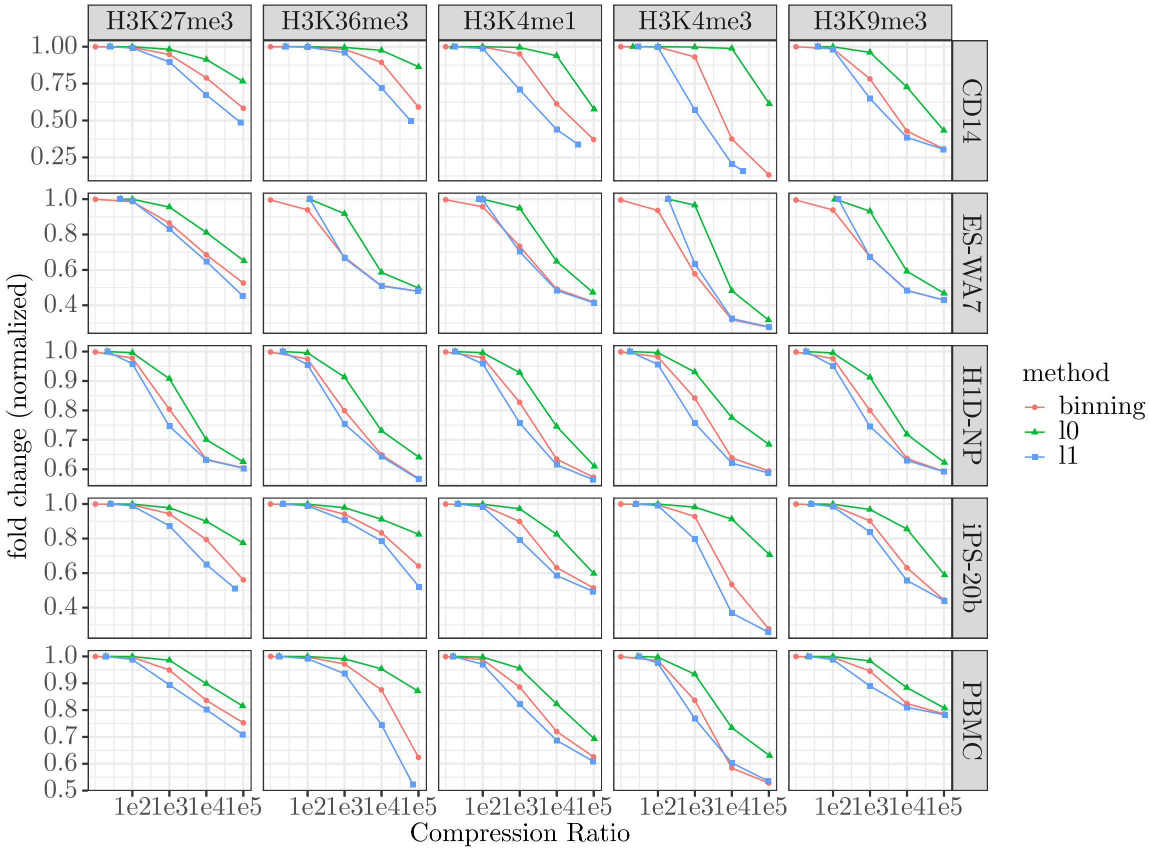

Figure S4: We selected A 10 million base pair section with the maximum number of MACS peaks for each cell line histone modification couple. Each signal within these sections is segmented, applying compression ratios of 10, 100, 1000, 10000, and 100000. We then calculate and plot the fold change comparing the mean signals over the peaks to the overall means, against the compression ratio. The fold change is normalized so that the maximum fold change is 1 for each cell line histone modification couple.

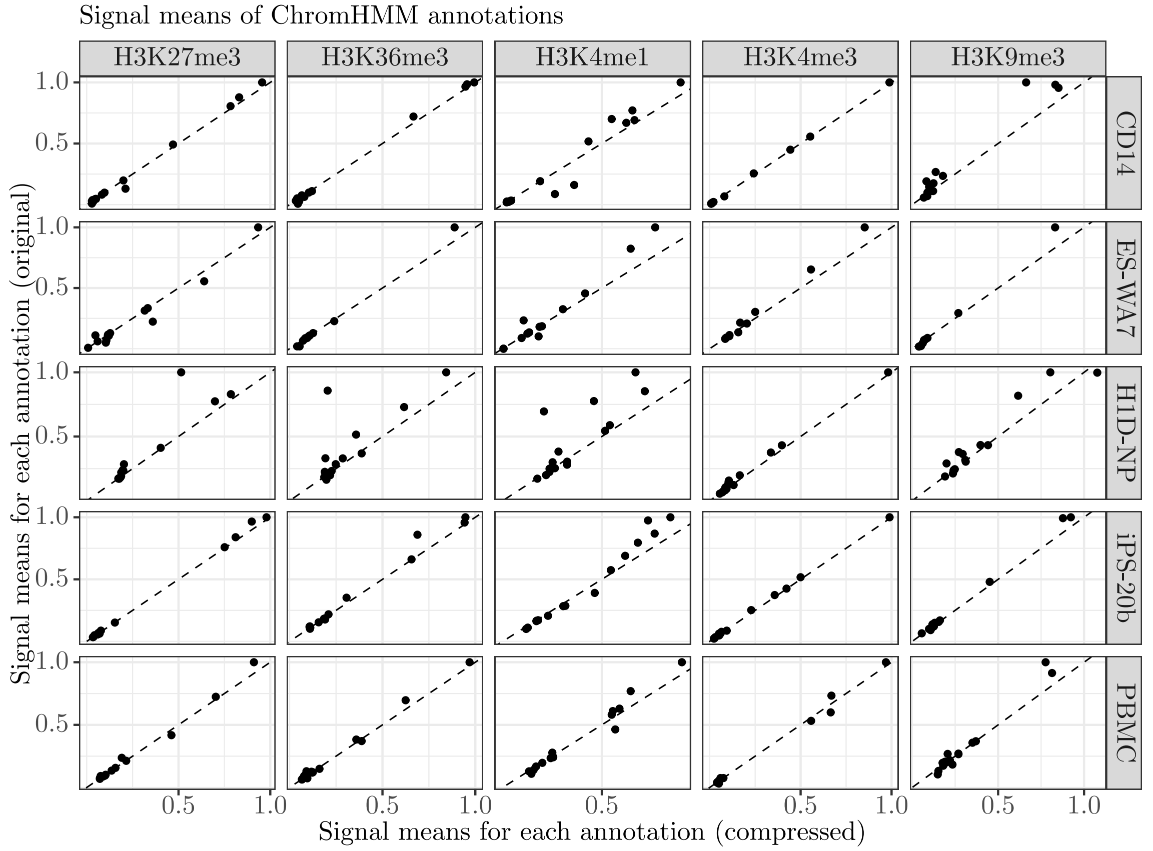

Figure S5: The second figure we included presents the findings of Project Roadmap, which analyzed 111 human epigenomes. As part of this study, ChromHMM, a state-of-the-art genome annotation tool employing a sophisticated HMM model, was utilized to segment these epigenomes into functional labels such as active/inactive TSS, promoters, enhancers, and gene bodies. We leveraged these segments to assess how effectively our algorithm preserves the characteristics of these annotations during compression. By comparing the means of various epigenetic signals over these labels before and after compression, with a fixed compression ratio of 10000 (where a 10 million base pair chromatin section is condensed into 1 thousand segments), we evaluated the fidelity of our compression approach.
